# Transcriptomic and Metabolomic Profiling Reveals the Antiproliferative Mechanism of Goose Serum and Plasma in SW1990 Cells

**DOI:** 10.3390/biology15100788

**Published:** 2026-05-15

**Authors:** Xiaolong Zhou, Mihan Wu, Han Wang, Xiangchen Li, Songbai Yang, Ayong Zhao

**Affiliations:** Key Laboratory of Applied Technology on Green-Eco-Healthy Animal Husbandry of Zhejiang Province, College of Animal Science and Technology, College of Veterinary Medicine, Zhejiang A & F University, 666 Wusu Road, Hangzhou 311300, China; zhouxiaolong@zafu.edu.cn (X.Z.);

**Keywords:** goose plasma, goose serum, metabolomic, SW1990 cells, transcriptome

## Abstract

Goose blood has anticancer properties and was recorded in ancient China, but the specific molecular mechanisms underlying this effect still require further exploration. In this study, SW1990 cells were treated with goose serum or plasma, and transcriptome analysis was performed to explore the function of goose blood on cancer cells. Metabolomic profiling was also performed on goose serum, goose plasma, chicken serum, and chicken plasma to identify the bioactive substances responsible for the anticancer effect. The study examined the effects of goose plasma and serum on SW1990 cells and compared the metabolites between goose and chicken blood. Wound scratch, CCK-8, and Annexin V-PI assays showed that goose plasma and serum inhibited SW1990 cell proliferation at 24 and 48 h. Both treatments reduced cell viability, with serum inducing early and late apoptosis and plasma inducing late apoptosis. RNA sequencing (RNA-seq) identified 2259 (1418 upregulated, 841 downregulated) and 2731 (1844 upregulated, 887 downregulated) differentially expressed genes (DEGs) in the plasma and serum groups versus the negative control (NC), respectively, and 689 DEGs between the plasma and serum groups. Gene Ontology (GO) and KEGG pathway analyses revealed that the DEGs were enriched in processes such as lipid metabolism, JAK-STAT, and IL-17 pathways. Untargeted liquid chromatography–tandem mass spectrometry (LC-MS/MS) analysis identified distinct metabolites in goose and chicken blood, with unique metabolites and differential ones between groups. In SW1990 cells, four metabolite subclusters matched the plasma and serum effects. In summary, goose blood can suppress cancer cells by regulating gene expression to affect the key signaling pathways involved in cancer cell apoptosis and autophagy. Certain metabolites present at high concentrations in goose blood, such as cucurbitacin D and Oleoyl-L-carnitine, may also contribute to the inhibition of cancer cell proliferation and migration. These findings suggest that goose blood holds broad application prospects as a future auxiliary drug for cancer treatment, and this study provides a theoretical basis for the further application of goose products.

## Simple Summary

This study investigated the anticancer properties of goose blood by treating SW1990 cancer cells with goose serum and plasma. Results showed that both treatments significantly inhibited cancer cell proliferation and viability, successfully inducing apoptosis. Transcriptome analysis revealed this suppression was driven by the regulation of genes involved in key pathways, including lipid metabolism, JAK-STAT signaling, and IL-17 signaling. In addition, metabolomic comparisons between goose and chicken blood identified unique bioactive substances in goose blood, such as cucurbitacin D, which likely contribute to halting cancer migration.

## 1. Introduction

Pancreatic ductal adenocarcinoma (PDAC) accounts for over 90% of pancreatic cancers and is projected to become the second leading cause of cancer-related deaths in the USA [1]. In 2025, an estimated 62,210 new cases and 49,830 deaths were expected, with a dismal 5-year survival rate of 11% overall and just 3% for metastatic cases [2]. Rising incidence rates (0.5–1.0% annually) and late-stage diagnoses (47% metastatic at detection) underscore its aggressive nature [3]. PDAC arises from precursor lesions such as pancreatic intraepithelial neoplasia (PanIN), intraductal papillary mucinous neoplasms (IPMNs), and mucinous cystic neoplasms (MCNs) [4]. Progression involves stepwise mutations, epithelial injury, and stromal interactions. The tumor microenvironment (TME) plays a critical role in suppressing immune responses, particularly in causing natural killer (NK) cell dysfunction, which enables immune evasion and treatment resistance [5]. The current treatment landscape consists of the following: Surgery: Curative for resectable tumors (10–15% of cases, median survival: 54.4 months) [4]. Chemotherapy: First-line therapies include modified FOLFIRINOX or gemcitabine nab paclitaxel (GnP), with GnP showing superior survival in the PASS-01 trial (median OS: 9.7 vs. 8.4 months) [5]. Targeted Therapies: Olaparib, a PARP inhibitor, is approved for BRCA-mutated PDAC. Radiation: Used for locoregional control in unresectable cases.

The blood of geese contains various cellular components, including heterophils (20.8%), lymphocytes (69.8%), eosinophils (17.6%), monocytes (1.5%), and basophils (1.5%) [6]. Commercial goose red blood cell preparations (at concentrations of 10% or 100%) have been used in vitro studies after removing plasma and white cells to retain red cell activity. Anticancer factors extracted from goose blood have shown significant effects in laboratory and clinical experiments. The inhibition rate against liver cancer cells reached 72.89%, and for sarcoma S180, it was 69.2%. These factors directly act on cancer cells, inhibiting their proliferation, leading to tumor shrinkage without toxic side effects. Recent research has found that bioactive components in the serum of White Lion geese can affect gastric cancer cells, but the specific mechanism remains unclear. Experiments suggest that these components may induce apoptosis or regulate cellular pathways [7]. Currently, goose blood preparations are limited to in vitro use, and future research should focus on isolating and purifying active components as well as developing standardized preparation processes.

Due to goose blood exhibiting anticancer activity with relatively mild side effects, it holds promise for development as a novel adjuvant anticancer drug. The aim of this study was to investigate the effect of goose serum and plasma on SW1990 cells. Transcriptome analysis of SW1990 cells was conducted to identify the genes and signaling pathways involved in the inhibition of cell proliferation by goose serum and plasma, and metabolomic profiling of goose serum and plasma was performed to explore the anticancer components of goose blood.

## 2. Materials and Methods

### 2.1. Ethics Statement

In this research, all experimental procedures involving animal experiments were carried out in accordance with the measures for the administration of experimental animals of Zhejiang Province (approved by the People’s Government of Zhejiang Province in 2009, promulgated by Order No. 263). All animal experiments in this study were approved by the Animal Protection and Use Committee of Zhejiang A&F University to ensure compliance with international animal welfare guidelines (Ethical license number: ZAFUAC202562).

### 2.2. Materials

The goose blood was collected from the Huzhou Taihu Zhuowang Goose Native Breed Farm. For each goose, 10 mL of blood was drawn from the wing vein, after which bleeding was stopped. To obtain the goose serum, the blood was placed in non-anticoagulant tubes and left to stand for 1–2 h. It was then centrifuged at 3000 rpm for 5 min to collect the supernatant (serum). To obtain goose plasma, the blood was collected in anticoagulant tubes and directly centrifuged at 3000 rpm for 5 min to remove the blood cells and collect the plasma. The resulting supernatants (serum or plasma) were then filtered through a 0.22 μm membrane for sterilization and stored at −80 °C. Chicken plasma and serum were obtained using the same procedure. Cucurbitacin D (MCE, HY-N1986, Monmouth Junction, NJ, USA) and Oleoyl-L-Carnitine (Aladdin, O356665, Shanghai, China) were also used to treat SW1990 cells.

### 2.3. Cell Culture

The SW1990 cell line is a widely used model in pancreatic cancer research, derived from human pancreatic adenocarcinoma epithelial cells from a spleen metastasis site [8,9]. The cell line is pivotal for studying the malignancy, proliferation, and metastasis of pancreatic adenocarcinoma cells. The human pancreatic cancer SW1990 cell line was cultured in Leibovitz’s L-15 medium (L-15, Nexell YC-3029, Shanghai, China) supplemented with 10% fetal bovine serum (FBS, ExCell FSP500, Montevideo, Uruguay) and 100 μg/mL penicillin/streptomycin dual-antibiotic solution. The cells were seeded in T25 culture flasks with airtight caps. During the culture process, the flasks were removed from the incubator once a day for ventilation purposes. During the experimental stage, Leibovitz’s L-15 medium containing 2% fetal bovine serum was used, while the control group was supplemented with PBS, and the experimental groups were treated with goose serum or plasma.

### 2.4. Real-Time Quantitative PCR Analysis

Primers for *NR4A1*, *NR4A2*, *NR4A3*, *MYC*, *TP53l11*, and *CASP10* were designed using Primer 6 software. Primers for actin beta (ACTB) were used as an internal control. Total RNA was extracted from cells using TRIzol^®^ Reagent (Invitrogen, Waltham, MA, USA) according to the manufacturer’s protocol. Reverse transcription of total RNA (1 μg) was performed using a RevertAid™ RT Reagent Kit (RR036A, Takara, Kyoto, Japan) in a 10 μL reaction volume according to the manufacturer’s instructions. Primer information for the real-time quantitative PCR (StepOne Plus Q3, Applied Biosystems, Waltham, MA, USA) is available in the supplemental information (Table S1).

### 2.5. Transcriptome Sequencing and Differentially Expressed Gene Analysis

Total RNA was extracted from cells using TRIzol^®^ Reagent (Invitrogen) according to the manufacturer’s protocol. The concentration and purity of the extracted RNA were assessed using a NanoDrop2000 (Thermo Fisher Scientific, Wilmington, DE, USA), the integrity of the RNA was evaluated by means of agarose gel electrophoresis, and the RQN value was determined using the Agilent 5300 (Santa Clara, CA, USA). The RNA samples were submitted for transcriptome sequencing by Shanghai Meiji Biomedical Technology Co., Ltd. (Shanghai, China) The reference genome was obtained from https://www.ncbi.nlm.nih.gov/genome/?term=human%5Borgn%5D (accessed on 16 October 2025).

Fragmentation buffer was used to fragment the RNA. Reverse transcription was performed to synthesize cDNA, followed by end repair, addition of an A-tail, and adapter ligation. DNA library amplification and quality assessment were then performed. Clean reads were obtained by removing unqualified reads from the original data and were aligned to the reference genome [10,11]. The expression levels of genes and transcripts were quantified separately using RSEM software (ver. 1.3.3, USA). Genes with a fold change ≥ 2 and adjusted *p*-value < 0.05 between samples were identified as differentially expressed genes (DEGs) [12,13]. Gene Ontology (GO) functional cluster analysis and Kyoto Encyclopedia of Genes and Genomes (KEGG) pathway enrichment analysis were performed. All raw data were deposited into the NCBI Sequence Read Archive (SRA) database (Accession Number: PRJCA039816).

### 2.6. Cell Scratch Healing Rate

SW1990 cells were placed in a 6-well plate and the inoculation density was adjusted to ensure that the cell monolayer reached 90–100% confluence. A sterile 200 μL pipette tip was used to create a vertical straight scratch in the center of the cell monolayer, maintaining consistent force; the number of scratches per well was kept the same, and the operation was completed quickly to minimize interference. The wells were rinsed slowly with PBS twice to remove the detached cells and debris from the scratch. A 2% FBS medium was added to avoid affecting cell proliferation. Initial scratch images were captured at 0 h using an inverted microscope, with marked imaging areas for subsequent imaging at 24 h or 48 h.

Cell migration rate analysis (based on scratch width): After the scratch experiment, photos were taken at 0, 24, and 48 h at the same position using an inverted microscope. The average distance between the two sides of the scratch (scratch width) was measured using Image J image processing software (Ver. 1.52p, USA). Three to five positions were selected in each well for measurement, and the average value was taken. The migration rate calculation formula is as follows: W0 h–W24 h/W0 h. All experiments were repeated three times; the results were expressed as mean ± standard deviation (SD). The differences were statistically analyzed using an independent sample *t*-test or one-way analysis of variance (ANOVA), and a *p*-value < 0.05 was considered statistically significant.

### 2.7. CCK-8 Assay

When the SW1990 cells were in the logarithmic growth phase, after digestion, they were resuspended in Dulbecco’s Modified Eagle Medium (DMEM) supplemented with 10% FBS. After counting, the cell density was adjusted to 2 × 10^4^ cells/mL. A volume of 100 μL was added to each well and inoculated into a 96-well plate. Each group was set up with 3 to 5 duplicate wells. Blank wells were filled with the same amount of medium as a blank control. After the cells adhered stably, the medium was replaced and the experimental treatment group was added. The cells were further cultured for 24 and 48 h at different time points. At each time point, the 96-well plate was taken out, and 10 μL of the CCK-8 working solution was added to each well, gently mixed, and then returned to the incubator for further incubation for 2 h. After incubation, the absorbance (OD_450_) of each well was read using an enzyme-linked spectrophotometer (BioTek Synergy H1 Hybrid Reader, Agilent, Santa Clara, CA, USA) at a wavelength of 450 nm. After subtracting the background of the blank well, the OD value reflects the number of viable cells and is used to draw the cell proliferation curve or calculate the inhibition rate.

### 2.8. Metabolomic Profiling Analysis

The serum and plasma samples from geese and chickens were collected from the wing vein. The serum or plasma was placed in a sterile conical tube and immediately frozen at −80 °C for further analysis. The samples were added to a 2 mL centrifuge tube, and 400 μL of extraction solution (methanol: water = 4:1 (*v*:*v*) containing 0.02 mg/mL of internal standard L-2-chlorophenylalanine was used for metabolite extraction. Samples were ground using the Wonbio-96c (Shanghai Wanbo Biotechnology Co., Ltd., Shanghai, China) frozen tissue grinder for 6 min (−10 °C, 50 Hz), followed by low-temperature ultrasonic extraction for 30 min (5 °C, 40 kHz). The samples were left at −20 °C for 30 min, centrifuged for 15 min (4 °C, 13,000× *g*), and the supernatant was transferred to the injection vial for LC-MS/MS analysis. Differential metabolites between the two groups were mapped into their biochemical pathways through metabolic enrichment and pathway analysis based on the KEGG database http://www.genome.jp/kegg/ (accessed on 18 October 2025). The Python (Ver. 3.13, USA) package “scipy.stats” https://docs.scipy.org/doc/scipy/ (accessed on 23 October 2025) was used to perform enrichment analysis to obtain the most relevant biological pathways for experimental treatments [14,15]. All raw data were deposited in the CNCB GSA database (accession number: OMIX010150).

### 2.9. Flow Cytometry

Apoptosis of SW1990 cells was detected by means of flow cytometry. Cells were stained using a fluorescein isothiocyanate annexin V apoptosis detection kit (CA1020; Solarbio, Beijing, China) in accordance with the manufacturer’s instructions. The cells were analyzed using a BD-FACS Canto-II flow cytometer (BD Biosciences, San Jose, CA, USA) and the percentage of apoptotic cells was quantified. Annexin V-fluorescein isothiocyanate-positive cells were classified as early apoptotic cells; PI-positive cells were classified as necrotic cells. Cells with both markers were classified as late apoptotic cells. All data were analyzed using FlowJo software (TreeStar FlowJo 10.4.0, Ashland, OR, USA).

### 2.10. Statistical Analysis

SPSS 20.0 (ver 20.0, SPAA Inc., Fort Collins, CO, USA) software was used for the statistical analysis of experimental data. The significant difference between the groups was analyzed via Student’s *t*-test or one-way analysis of variance (ANOVA). Data were expressed as mean ± standard error of the mean (SEM) and *p* < 0.05 indicated a statistically significant difference. The correlation between the differential microbes and differential metabolites was determined using Spearman’s correlation coefficient.

## 3. Results

### 3.1. Effect of Goose Plasma or Serum on the Proliferation of SW1990 Cells

**Figure 1:**
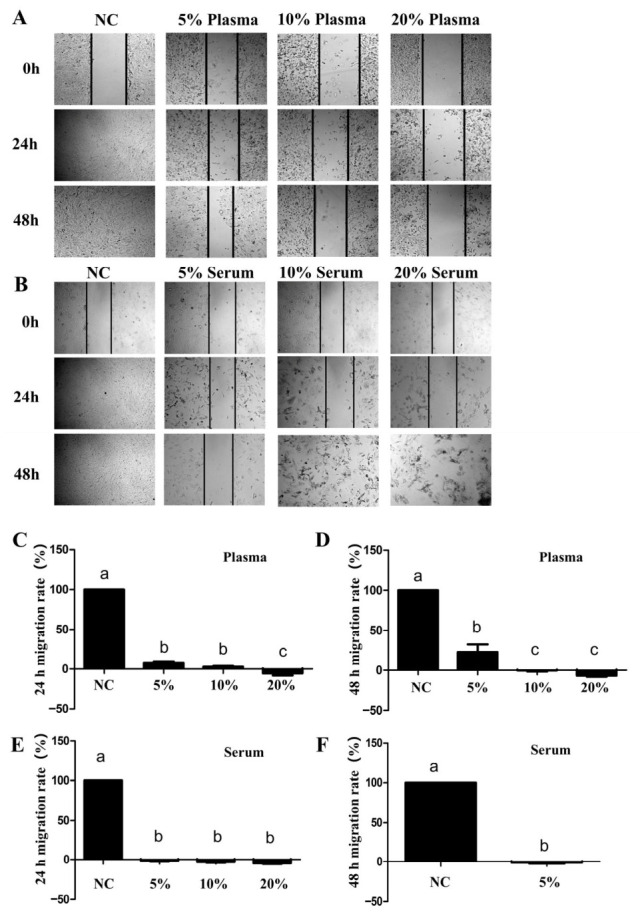
The effect of goose plasma and serum on the migration of SW1990 cells: (**A**) Scratch test of the SW1990 cells treated with goose plasma (*n* = 3); (**B**) scratch test of the SW1990 cells treated with goose serum (*n* = 3); (**C**) the migration rate of SW1990 cells at 24 h treated with goose plasma; (**D**) the migration rate of SW1990 cells at 48 h treated with goose plasma; (**E**) the migration rate of SW1990 cells at 24 h treated with goose serum; and (**F**) the migration rate of SW1990 cells at 48 h treated with goose serum. Different lowercase letters represent *p* < 0.05.

To investigate the effect of goose blood on the migration of SW1990 cells, a wound scratch assay was performed. The results showed that goose plasma significantly inhibited the proliferation of SW1990 cells at concentrations of 5%, 10%, and 20% at both 24 and 48 h after treatment (Figure 1A,C,D). Goose serum also significantly suppressed the migration of SW1990 cells; notably, treatment with 10% and 20% goose serum led to cell death at 48 h after treatment (Figure 1B,E,F).

**Figure 2:**
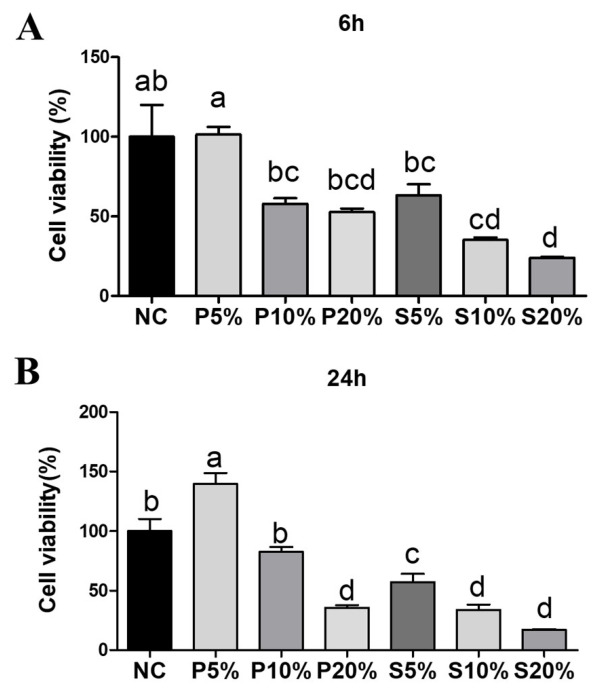
Cell viability analysis of SW1990 cells treated with goose blood: (**A**) CCK-8 analysis of SW1990 cells at 6 h after treatment with goose plasma or serum (*n* = 8). P5% = 5% plasma; S5% = 5% serum. (**B**) CCK-8 analysis of SW1990 cells at 24 h after treatment with goose plasma or serum (*n* = 8). Different lowercase letters represent *p* < 0.05.

To further verify the effects of goose blood on SW1990 cell proliferation, a CCK-8 assay was performed. The results showed that the cell viability of SW1990 cells was significantly decreased by both goose plasma and goose serum (Figure 2A,B).

**Figure 3:**
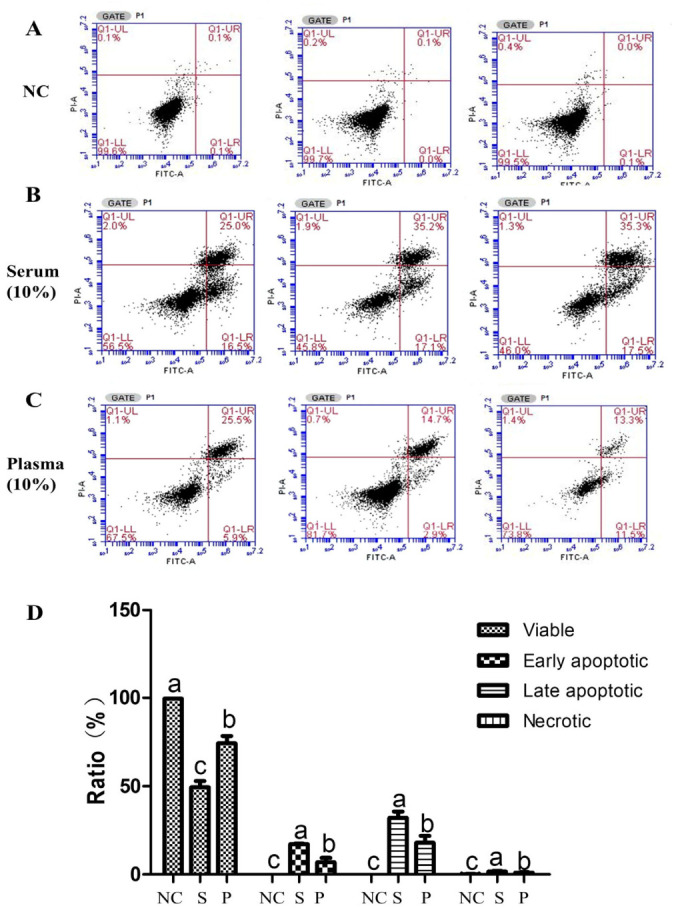
Effects of goose plasma and serum on the apoptosis of SW1990 cells: (**A**) Annexin V and PI analysis on the SW1990 cells treated with pbs (*n* = 3); (**B**) annexin V and PI analysis on the SW1990 cells treated with 10% goose serum (*n* = 3); (**C**) annexin V and PI analysis on the SW1990 cells treated with 10% goose plasma (*n* = 3); and (**D**) the ratio of goose plasma and serum on early apoptosis and late apoptosis. Different lowercase letters represent *p* < 0.05. NC = negative control, S = serum, and P = plasma.

Based on these results, 10% serum and 10% plasma were selected for the treatment of SW1990 cells in subsequent experiments. An Annexin V-PI assay was also performed to detect the effect of goose serum and plasma on the apoptosis of SW1990 cells. The results showed that both 10% goose serum and 10% goose plasma significantly reduced the viability of SW1990 cells by promoting both early and late apoptosis. Moreover, the proportion of early and late apoptotic cells in the 10% serum group was significantly higher than that in the 10% plasma group (Figure 3A,D).

### 3.2. Differentially Expressed Genes Induced by Goose Plasma or Serum Treatment in SW1990 Cells

To investigate the differences in mRNA expression among the three treatment conditions, RNA-seq was performed on SW1990 cells treated with goose serum or plasma. The NC group consisted of PBS-treated SW1990 cells. From the RNA-seq results, a total of approximately 56.05 billion raw bases (371.22 million raw reads) and 55.25 billion clean bases (368.15 million clean reads) were obtained. The clean data were aligned to the Homo sapiens reference genome (GCF_000001405.40), with mapping rates ranging from 96.93% to 97.26%. A total of 33,814 genes were detected, comprising 33,158 known genes and 656 novel genes. The Q20 (percentage of bases with a sequencing quality score above 98.74%) and Q30 values of each sample exceeded 96.07%, indicating high-quality sequencing data suitable for subsequent analysis (Table S2). Gene functional annotations are provided in Table S3, and gene expression data can be found in Table S4.

**Figure 4:**
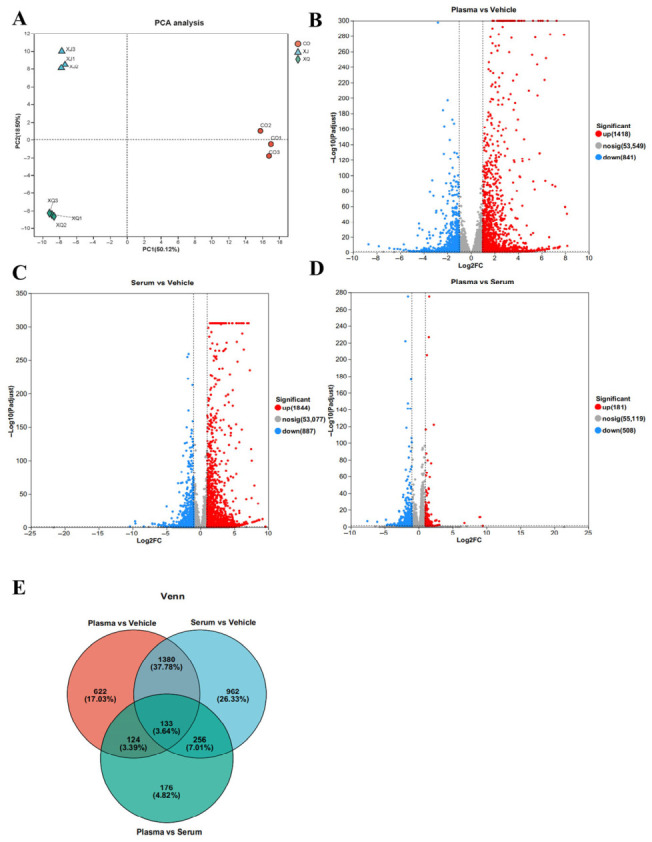
Volcano plot of DEGs between different groups: (**A**) PCA analysis of different groups; (**B**) DEGs between goose plasma and NC groups (*n* = 3); (**C**) DEGs between goose serum and NC groups; (**D**) DEGs between goose plasma and serum groups; and (**E**) Venn diagram of DEGs among goose plasma, goose serum, and NC groups.

Principal component analysis (PCA) was performed; the first two principal components captured the majority of the variation among samples, indicating that the results were highly representative and the conclusions drawn were reliable (Figure 4A). In the goose plasma treatment group compared with the NC group, 1418 upregulated and 841 downregulated DEGs were identified at 24 h (Figure 4B, Table S5). In the goose serum treatment group, 1844 upregulated and 887 downregulated DEGs were identified at 24 h (Figure 4C, Table S6). When comparing the goose plasma treatment group directly with the goose serum treatment group, 181 upregulated and 508 downregulated DEGs were found at 24 h (Figure 4D, Table S7). Notably, overlapping DEGs were observed among the different group comparisons after treatment with goose plasma and goose serum; a total of 1503 genes (1380 + 133) were commonly differentially expressed between the plasma vs. NC and serum vs. NC comparisons. Additionally, 746 unique DEGs (622 + 124) were identified in the plasma vs. NC comparison at 24 h, and 1218 unique DEGs (962 + 256) were identified in the serum vs. NC comparison at 24 h (Figure 4E).

### 3.3. Gene Expression Pattern Analysis for Goose Plasma and Serum Treatment in SW1990 Cells

GO enrichment analysis showed that, compared with the NC group, upregulated DEGs in the goose plasma treatment group were mainly enriched in the regulation of cellular processes and biological regulation (Figure S1A, Table S8), whereas downregulated DEGs were mainly enriched in lipid metabolic processes, mitotic cell cycle processes, cytoskeleton organization, and sterol biosynthetic processes (Figure S1B, Table S9). In the serum treatment group compared with the NC group, upregulated DEGs were mainly enriched in the regulation of signal transduction, response to stimulus, and regulation of cell communication (Figure S1C, Table S10), while downregulated DEGs were mainly enriched in the regulation of cellular processes, biological regulation, regulation of biological processes, and developmental processes (Figure S1D, Table S11). When comparing the plasma group with the serum group, upregulated DEGs were mainly enriched in the regulation of developmental processes, DNA-binding transcription factor activity, and regulation of biological processes (Figure S1E, Table S12), whereas downregulated DEGs were mainly enriched in response to biotic stimulus, lipid metabolic processes, and steroid metabolic processes (Figure S1F, Table S13).

**Figure 5:**
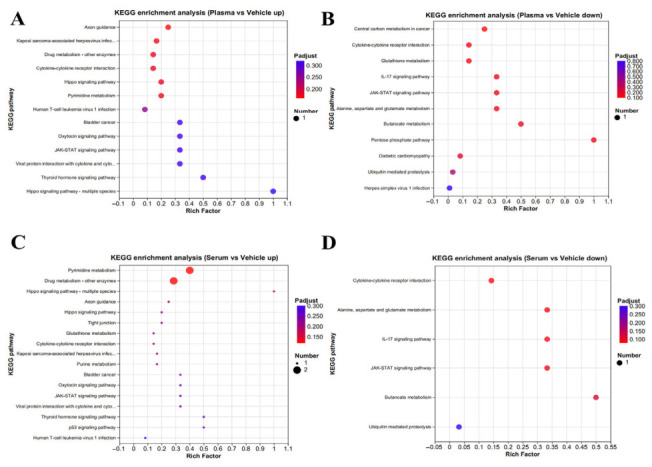
Top 20 KEGG enrichment analyses: (**A**) Top 20 KEGG enrichment analysis of the upregulated genes between the goose plasma and NC groups; (**B**) top 20 KEGG enrichment analysis of the downregulated genes between the goose plasma and NC groups; (**C**) top 20 KEGG enrichment analysis of the upregulated genes between the goose serum and NC groups; and (**D**) top KEGG0 GO enrichment analysis of the downregulated genes between the goose serum and NC groups.

KEGG pathway enrichment analysis was also performed. Compared with the NC group, upregulated DEGs in the plasma treatment group were mainly enriched in axon guidance, cytokine–cytokine receptor interaction, the Hippo signaling pathway, pyrimidine metabolism, the oxytocin signaling pathway, and the JAK-STAT signaling pathway (Figure 5A, Table S14), whereas downregulated DEGs were mainly enriched in central carbon metabolism in cancer, cytokine–cytokine receptor interaction, glutathione metabolism, the IL-17 signaling pathway, the JAK-STAT signaling pathway, alanine, aspartate, and glutamate metabolism, butanoate metabolism, and the pentose phosphate pathway (Figure 5B, Table S15). In the serum treatment group compared with the NC group, upregulated DEGs were mainly enriched in pyrimidine metabolism and drug metabolism (Figure 5C, Table S16), while downregulated DEGs were mainly enriched in cytokine–cytokine receptor interaction, alanine, aspartate and glutamate metabolism, the IL-17 signaling pathway, the JAK-STAT signaling pathway, and butanoate metabolism (Figure 5D, Table S17). When comparing the plasma group with the serum group, no significantly enriched pathways were identified among either upregulated or downregulated DEGs. Taken together, these results showed that, in the plasma-treated group, downregulated DEGs (not upregulated DEGs) were involved in central carbon metabolism in cancer and the IL-17 signaling pathway, whereas the JAK-STAT pathway was regulated by both upregulated and downregulated DEGs. Similarly, in the serum-treated group, downregulated DEGs (not upregulated DEGs) were involved in the IL-17 signaling pathway, and the JAK-STAT pathway was again regulated by both upregulated and downregulated DEGs.

**Figure 6:**
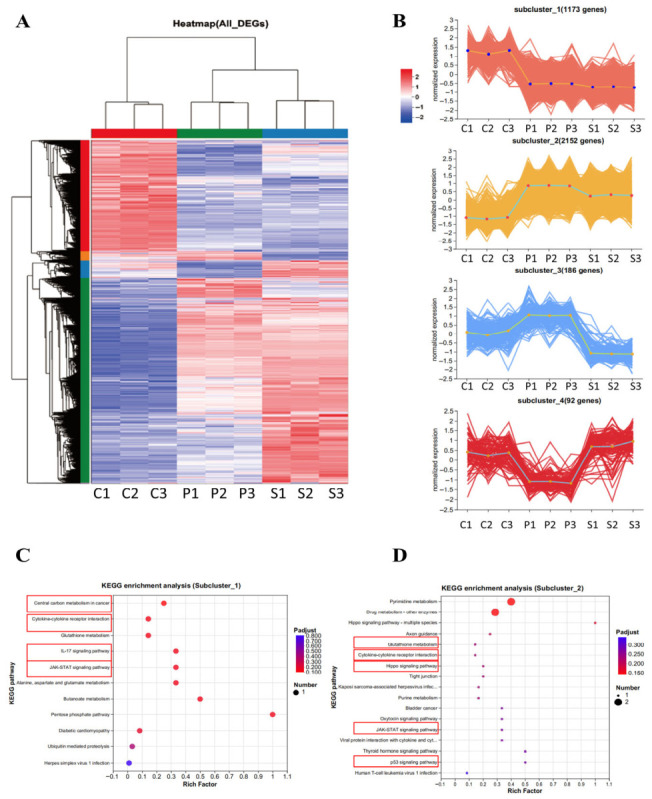
DEG expression pattern for sw1990 cells treated with goose plasma and serum: (**A**) Heat map of DEGs, *X*-axis: C1–C3 = negative control group, P1–P3 = plasma group, and S1–S3 = serum group; (**B**) four subclusters of cluster analysis. *Y*-axis: log10 (expression); (**C**) KEGG enrichment analysis of DEGs in subcluster_1; and (**D**) KEGG enrichment analysis of DEGs in subcluster_2.

To identify potential expression trends among the groups, a cluster analysis was performed on all DEGs, which were classified into four subclusters based on gene expression (Figure 6A). Since the effects of plasma and serum on SW1990 cells are consistent, we focused on genes that exhibited a consistent expression pattern between the plasma and serum groups. We found that subcluster_1 and subcluster_2 fit this expression pattern (Figure 6B, Tables S18–S21). KEGG enrichment analysis of subcluster_1 revealed that its DEGs were mainly enriched in central carbon metabolism in cancer, cytokine–cytokine receptor interaction, glutathione metabolism, the JAK-STAT signaling pathway, alanine, aspartate and glutamate metabolism, butanoate metabolism, the pentose phosphate pathway, and the IL-17 signaling pathway (Figure 6C, Table S22). KEGG enrichment analysis of subcluster_2 revealed that its DEGs were primarily enriched in pyrimidine metabolism, the Hippo signaling pathway, and the JAK-STAT signaling pathway (Figure 6D, Table S23).

**Figure 7:**
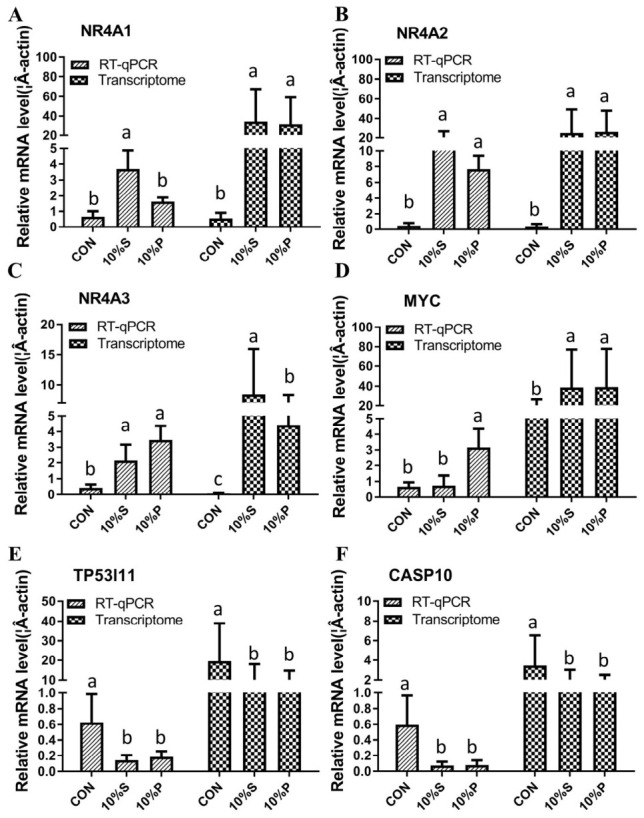
Validation of the transcriptome using RT-qPCR: (**A**) *NR4A1* mRNA levels following treatment with 10% serum or 10% plasma, *n* = 3; (**B**) *NR4A2* mRNA levels following treatment with 10% serum or 10% plasma, *n =* 3; (**C**) *NR4A3* mRNA levels following treatment with 10% serum or 10% plasma, *n =* 3; (**D**) *MYC* mRNA levels following treatment with 10% serum or 10% plasma, *n* = 3; (**E**) *TP53I11* mRNA levels following treatment with 10% serum or 10% plasma, *n* = 3; and (**F**) *CASP10* mRNA levels following treatment with 10% serum or 10% plasma, *n* = 3. Different lowercase letters represent *p* < 0.05.

The expression of genes related to pancreatic cancer was validated against the transcriptome results. We found that the genes *NR4A1*, *NR4A2*, *NR4A3*, *CASP10*, *TP53I11*, and *MYC* showed consistent expression trends between RT-qPCR and RNA-seq results (Figure 7A–E).

### 3.4. Compositions and Differential Untargeted Metabolites in the Goose and Chicken Blood

**Figure 8:**
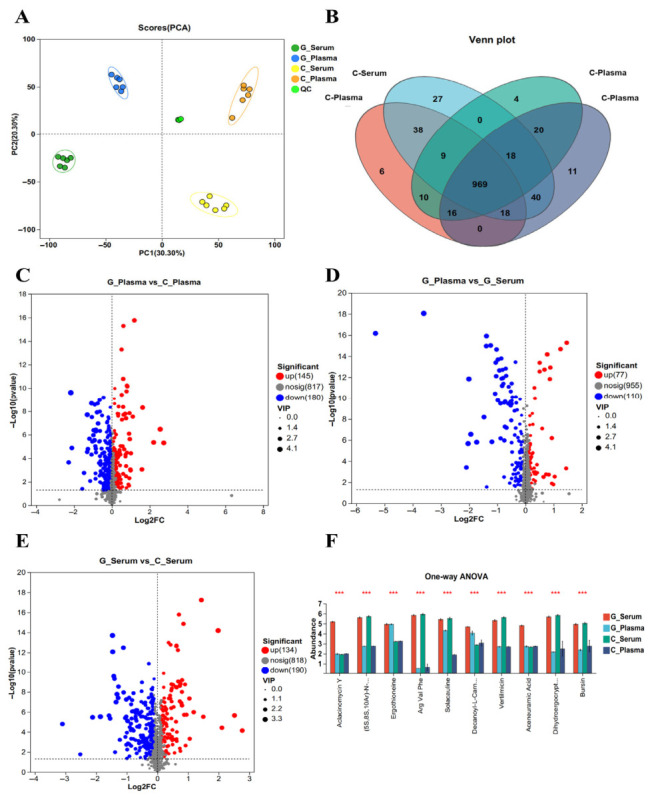
Differences in metabolites between groups: (**A**) The PCA scores among goose serum (G_serum), goose (G_plasma), chicken serum (C_serum), and chicken plasma (C_plasma) groups (*n* = 6); (**B**) Venn diagram of metabolites among goose serum, goose plasma, chicken serum, and chicken plasma groups; (**C**) DMs between goose plasma and chicken plasma groups; (**D**) DMs between goose plasma and goose serum groups; (**E**) DMs between goose serum and chicken serum groups; and (**F**) DMs among four groups. *** *p* < 0.001.

Given that goose blood can suppress the proliferation and migration of SW1990 cells, whereas chicken blood cannot, untargeted LC-MS/MS analysis was performed on goose and chicken plasma and serum to identify potential bioactive substances in goose blood responsible for its anticancer activity. PCA revealed significant differences in metabolite abundance patterns among the goose plasma, goose serum, chicken plasma, chicken serum, and quality control (QC) groups. Greater separation indicated more distinct metabolite profiles between sample groups (Figure 8A). The results demonstrated clear separation of metabolites among the groups. A Venn diagram was used to display the numbers of common and unique metabolites across the samples; the number of unique metabolites in the goose plasma, goose serum, chicken plasma, and chicken serum groups was 4, 11, 6, and 27, respectively (Figure 8B).

A total of 145 differential metabolites (DMs) were upregulated and 180 DMs were downregulated between the goose plasma and chicken plasma groups (Figure 8C, Table S24). Between the goose plasma and goose serum groups, 77 DMs were upregulated and 110 DMs were downregulated (Figure 8D, Table S25). Between the goose serum and chicken serum groups, 134 DMs were upregulated and 190 DMs were downregulated (Figure 8E, Table S26). A one-way ANOVA was performed among the four groups, and the results showed that levels of aclacinomycin Y and aceneuramic acid in goose serum were significantly higher than those in the other groups, whereas the levels of ergothioneine and decanoyl-L-carnitine in both goose serum and goose plasma were significantly higher than those in chicken serum and plasma (Figure 8F, Table S27).

### 3.5. Differential Metabolites Analysis for Goose Plasma, Goose Serum, Chicken Plasma, and Serum

**Figure 9:**
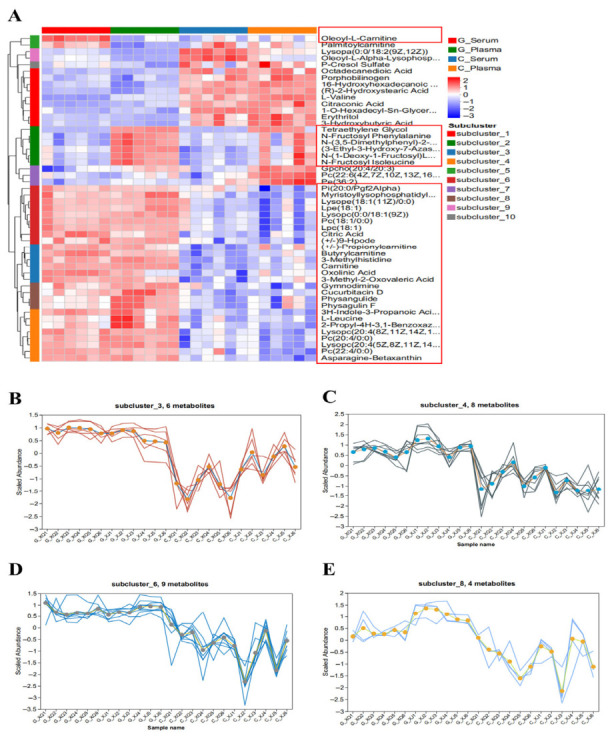
Differential metabolites analysis between goose blood and chicken blood: (**A**) Heat map of differential metabolites among the goose plasma, goose serum, chicken plasma, and chicken serum groups; (**B**) Subcluster_3 of differential metabolites among the groups. G_XQ = goose serum, G_XJ = goose plasma, C_XQ = chicken serum, and C_XJ = chicken plasma; (**C**) Subcluster_4 of differential metabolites among the groups; (**D**) Subcluster_6 of differential metabolites among the groups; and (**E**) Subcluster_8 of differential metabolites among the groups.

Variations in metabolite composition and abundance were shown in the heat map (Figure 9A, Table S27). The metabolites were divided into 10 subclusters based on their enrichment patterns. Since the effects of plasma and serum on SW1990 cells were consistent, we focused on DMs that exhibited a consistent expression pattern between the plasma and serum groups. We found that subcluster_3, subcluster_4, subcluster_6, and subcluster_8 fit these expression patterns. In subcluster_3, the metabolites included oxolinic acid, butyrylcarnitine, (±)-propionylcarnitine, 3-methylhistidine, carnitine, and 3-methyl-2-oxovaleric acid. In subcluster_4, the metabolites included L-leucine, 3H-indole-3-propanoic acid, 2-propyl-4H-3,1-benzoxazin-4-one, PC (22:4/0:0), asparagine-betaxanthin, LysoPC (20:4(8Z,11Z,14Z,17Z)/0:0), and LysoPC (20:4(5Z,8Z,11Z,14Z)/0:0). In subcluster_6, the metabolites included PC (18:1/0:0), LysoPC (0:0/18:1(9Z)), myristoyllysophosphatidylcholine, LPC (18:1), PI (20:0/PGF2α), LysoPE (18:1(11Z)/0:0), citric acid, LPE (18:1), and (±)-9-HpODE. In subcluster_8, the metabolites included physangulide, physagulin F, cucurbitacin D, and gymnodimine (Figure 9B–E, Tables S28–S37).

**Figure 10:**
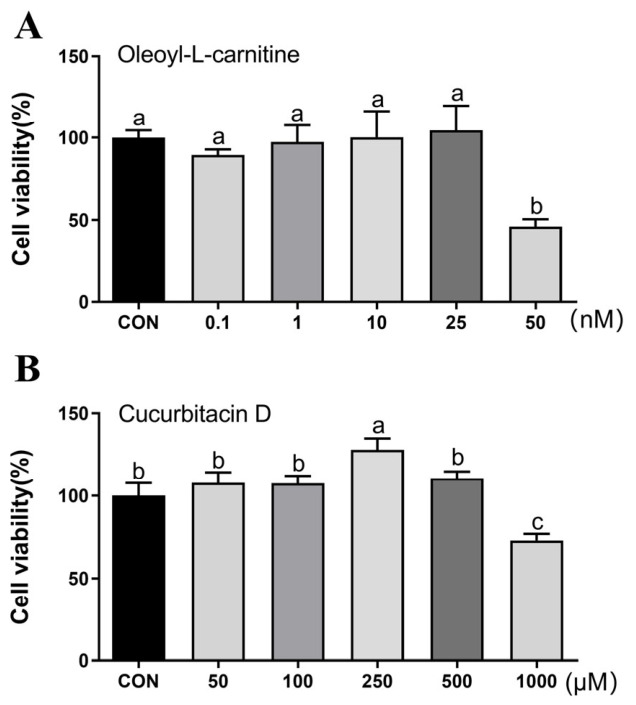
Cell viability analysis of SW1990 cells treated with differential metabolites: (**A**) CCK-8 analysis of SW1990 cells treated with Oleoyl-L-carnitine (*n* = 8) and (**B**) CCK-8 analysis of SW1990 cells treated with Cucurbitacin D (*n* = 8). Different lowercase letters represent *p* < 0.05.

We found that oleoyl-L-Carnitine was enriched in goose serum, while cucurbitacin D was enriched in both goose plasma and serum. To verify the effects of oleoyl-L-Carnitine and cucurbitacin D on SW1990 cells, a CCK-8 assay was performed. The results showed that 50 nM oleoyl-L-Carnitine can significantly suppress the proliferation of SW1990 cells (Figure 10A), and 1000 μM cucurbitacin D can significantly suppress the proliferation of SW1990 cells (Figure 10B).

## 4. Discussion

Goose blood is a natural medicinal resource in China that has shown unique promise in both traditional applications and modern research. In recent years, with the continuous advancement of medical research, the application prospects of goose blood in the field of cancer treatment have gradually become a research hotspot. Modern scientific research has found that goose blood contains various active components, including immunoglobulins, enzymes, and polysaccharides. These components may exert anticancer effects through multiple mechanisms. On one hand, the immunologically active substances in goose blood can activate the human immune system and enhance the activity and abundance of immune cells such as macrophages and T lymphocytes, enabling the body to more effectively identify and eliminate tumor cells, thereby improving its anti-tumor immune response. On the other hand, some components may induce tumor cell apoptosis and inhibit tumor angiogenesis, effectively curbing tumor growth and metastasis of tumors at their source. In the future, with further research and continuous technological innovation, goose blood is expected to become a new force in the field of cancer treatment, bringing new hope to cancer patients.

Although the major components of goose serum and plasma are largely the same, they differ in preparation and composition. Plasma is the liquid portion of blood obtained by centrifuging blood samples collected into tubes containing anticoagulants, which prevent blood clotting; therefore, plasma still contains fibrinogen and other clotting factors. Serum is obtained by allowing blood to clot naturally and then centrifuging the sample to remove the clot, and consequently lacks clotting factors like fibrinogen. In studies of geese, plasma is used to measure various biochemical and physiological parameters, including total protein, albumin, globulin, urea, uric acid, creatinine, hormones (e.g., growth hormone, insulin-like growth factor-1), immune indices (e.g., immunoglobulins), and antioxidant enzymes. These analyses require plasma because it retains clotting proteins and other soluble factors that are important for physiological assessment [16,17]. Serum is often used for amino acid profiling and other biochemical analyses in which clotting factors are not needed or might interfere. For example, in geese, serum amino acid profiles have been studied by collecting blood without an anticoagulant and analyzing serum amino acids such as valine, aspartic acid, arginine, citrulline, glycine, and ornithine.

Goose serum from White Lion geese has been shown to significantly inhibit the proliferation of human gastric cancer AGS cells by disrupting the cell cycle and promoting apoptosis [2]. Goose serum was found to reduce cancer cell viability and migration capacity in a dose- and time-dependent manner. In this study, we observed that both goose serum and goose plasma could inhibit the migration and proliferation of SW1990 cells, and this inhibition was also dose- and time-dependent (Figure 1A–F). Previous studies have found that goose serum can induce apoptosis in gastric cancer cells, with the apoptosis rate increasing alongside a decrease in viable cell numbers, and both early and late apoptotic processes were observed. In the present study, we found that goose serum inhibited the viability of SW1990 cells by promoting both early and late apoptosis, whereas goose plasma suppressed the viability of SW1990 cells by promoting late apoptosis only (Figure 2).

To explain why serum exhibits a stronger inhibitory effect on SW1990 cell proliferation than plasma, we examined the metabolomic differences between serum and plasma. Two pivotal lipid metabolites, oleoyl-L-Carnitine and palmitoylcarnitine, were significantly enriched in serum compared to plasma. As key acylcarnitine derivatives, these molecules participate in the regulation of cellular fatty acid β-oxidation and energy metabolism reprogramming in tumor cells. Elevated levels of oleoyl-L-Carnitine and palmitoylcarnitine can suppress the abnormal proliferation of pancreatic cancer cells by interfering with lipid energy supply and inducing metabolic stress.

Mechanistically, serum is obtained after blood coagulation and the removal of fibrinogen and clotting factors, which relatively concentrates bioactive small-molecule metabolites such as acylcarnitines. In contrast, plasma retains the complete coagulation protein system, which may dilute or antagonize the biological activity of these functional metabolites. Therefore, the higher abundance of oleoyl-L-Carnitine and palmitoylcarnitine in serum largely accounts for its superior inhibitory effect on SW1990 cell growth compared to plasma.

The RNA-seq results revealed significantly enriched pathways. In the plasma treatment group, the upregulated gene *RASSF6* was involved in the Hippo signaling pathway, the upregulated gene *IL24* was involved in the JAK-STAT signaling pathway and cytokine–cytokine receptor interaction, and the downregulated gene *IL17RE* was involved in cytokine–cytokine receptor interaction and the IL-17 signaling pathway. The downregulated gene *PIAS3* was involved in ubiquitin-mediated proteolysis and the JAK-STAT signaling pathway, and the downregulated gene *G6PD* was involved in central carbon metabolism in cancer, glutathione metabolism, and the pentose phosphate pathway. In the serum treatment group, the upregulated gene *RASSF6* was similarly involved in the Hippo signaling pathway; the upregulated gene *RRM2B* was involved in glutathione metabolism and the p53 signaling pathway; and the upregulated gene *IL24*, as observed in the plasma treatment, was involved in cytokine–cytokine receptor interaction and the JAK-STAT signaling pathway. The downregulated gene *IL17RE* was also, as in the plasma treatment, involved in cytokine–cytokine receptor interaction and the IL-17 signaling pathway, and the downregulated gene *PIAS3* was similarly involved in ubiquitin-mediated proteolysis and the JAK-STAT signaling pathway.

Notably, two subclusters with opposite expression patterns emerged from the RNA-seq cluster analysis. In the RNA-seq clusters, genes in subcluster_1 were downregulated by goose plasma and serum, while genes in subcluster_2 were upregulated by goose plasma and serum. These results indicated that goose plasma and serum inhibited SW1990 cells and suppressed cell migration through downregulated genes via the IL-17 signaling pathway (*IL17RE*), central carbon metabolism in cancer (*G6PD*), cytokine–cytokine receptor interaction (*IL17RE*), and the JAK-STAT signaling pathway (*PIAS3*); and through upregulated genes via the p53 signaling pathway and glutathione metabolism (*RRM2B*), the Hippo signaling pathway (*RASSF6*), and the JAK-STAT signaling pathway (*IL24*).

The gene *IL17RE* encodes a transmembrane protein that functions as a receptor for the cytokine interleukin-17C (IL-17C). IL-17RE, together with IL-17RA, forms a heterodimeric receptor complex through which IL-17C signals. This signaling pathway plays a critical role in regulating early innate immunity, particularly in epithelial cells, and in promoting inflammatory responses [18,19,20]. *IL17RE* contributes to cancer primarily through its function as the receptor for IL-17C, which participates in inflammatory and immune pathways that can influence tumor biology [21]. IL-17C/IL-17RE signaling has been implicated in promoting tumor growth, invasion, and immune evasion in several cancer types by activating pro-inflammatory pathways such as NF-κB and MAPK. This pathway may also enhance the function of Th17 cells, which can contribute to tumor-promoting inflammation [21]. However, IL-17 signaling can have dual roles. In some contexts, it may enhance anti-tumor immune responses by recruiting cytotoxic T lymphocytes (CTLs) and natural killer T (NKT) cells. The overall impact of *IL-17RE* in cancer likely depends on tumor type, microenvironment, and immune context [22,23]. In this study, we found that goose plasma and serum could suppress the IL-17 signaling pathway and cytokine–cytokine receptor interaction by reducing the expression of the gene *IL-17RE*.

Glucose-6-phosphate dehydrogenase (*G6PD*) plays a significant role in cancer through its function as a rate-limiting enzyme in the pentose phosphate pathway (PPP). This enzyme supports cancer cell proliferation and survival by enabling metabolic reprogramming that fuels rapid growth and resistance to oxidative stress [24]. *G6PD* promotes tumor cell proliferation by producing NADPH and ribose-5-phosphate via the PPP. NADPH maintains redox balance by detoxifying reactive oxygen species (ROS), thereby supporting antioxidant defenses essential for cancer cell survival under conditions of oxidative stress [25,26,27]. *G6PD* is linked to the activation of pathways such as STAT3, enhancing epithelial–mesenchymal transition (EMT) and tumor invasion; it can also maintain DNA synthesis and repair capacity, supporting tumor development and therapy resistance [28,29]. *G6PD* is overexpressed in many cancer types (hepatocellular carcinoma, pancreatic cancer, and breast cancer), with higher levels often correlating with poor prognosis [30]. In this study, we found that goose plasma and serum could suppress central carbon metabolism in cancer by reducing the expression of *G6PD*.

*PIAS3* (Protein Inhibitor of Activated STAT3) acts as a canonical negative regulator of the STAT3 signaling pathway and plays a vital role in modulating cancer cell proliferation, apoptosis and immune response. *PIAS3* suppresses STAT3 transcriptional activity by blocking its DNA-binding capacity, thereby restraining oncogenic STAT3 signaling and exerting tumor-suppressive roles [31,32,33]. In the present study, however, the expression of *PIAS3* was downregulated following goose serum and plasma intervention. Notably, this reduced *PIAS3* level does not contribute to the observed anticancer effect. The anti-proliferative function mediated by the JAK-STAT pathway is primarily driven by the modulation of core JAK-STAT signaling activity and downstream target genes, rather than dependent on *PIAS3* upregulation. The decreased *PIAS3* expression is considered an independent molecular alteration and does not conflict with the overall JAK-STAT-mediated anticancer regulation.

*RRM2B* (Ribonucleotide Reductase M2 B) is critically involved in cancer progression via regulating DNA replication, damage repair and oxidative stress homeostasis. As a p53-inducible subunit of ribonucleotide reductase, *RRM2B* supplies deoxyribonucleotides for DNA synthesis and genomic stability maintenance, and its overexpression is well documented to support cancer cell survival under oxidative stress. Elevated *RRM2B* commonly enhances tumor resistance to chemo- and radiotherapy by reducing DNA damage and apoptosis [34,35,36]. In this study, *RRM2B* expression was significantly upregulated following goose serum and plasma intervention. Considering the pro-survival role of *RRM2B*, this upregulation is not contradictory to the anticancer phenotype. Instead, the increased *RRM2B* is regarded as a compensatory cellular stress response: serum/plasma treatment induced proliferation suppression and cellular stress, triggering feedback upregulation of *RRM2B* to initiate endogenous DNA repair and stress defense. This passive self-protective upregulation could not reverse the overall inhibitory effect on cell proliferation, indicating that the altered *RRM2B* acts only as a bystander compensatory change and is not functionally associated with the anticancer action of goose serum and plasma.

*RASSF6* (Ras association domain family member 6) functions as a tumor suppressor in cancer by regulating apoptosis, cell cycle arrest, and cell proliferation through multiple mechanisms involving both Hippo signaling-dependent and -independent pathways [37,38]. *RASSF6* expression is frequently reduced in various human cancers, including gastric, pancreatic, colorectal, breast, bladder, and melanoma, with low levels correlating with poor prognosis. *RASSF6* can suppress tumor growth and invasion, and its overexpression inhibits colony formation and proliferation in cancer cells. Mechanistically, *RASSF6* blocks MDM2-mediated degradation of the tumor suppressor p53, enhancing its pro-apoptotic functions. Additionally, *RASSF6* influences the expression of tumor suppressor genes like *p16INK4A* and *p14ARF* by suppressing *BMI1* expression [39,40]. The Hippo pathway is a crucial tumor-suppressive signaling cascade that controls cell proliferation and apoptosis primarily through MST1/2 and LATS1/2 kinases and their downstream effectors. RASSF6 binds to MST1/MST2 kinases and inhibits their activity, thereby antagonizing Hippo pathway signaling [41].

IL24 signals canonically through binding to IL-20 receptor complexes (IL-20R1/IL-20R2 and IL-22R/IL-20R2), activating JAK family kinases (JAK1, JAK3, Tyk2) and downstream STAT transcription factors STAT1 and STAT3 in epithelial cells. However, in cancer cells, IL24 can induce apoptosis independently of the JAK-STAT pathway, especially at high concentrations. IL24 also activates SOCS proteins involved in the negative regulation of JAK-STAT signaling and inflammatory responses, linking it to both JAK-STAT-dependent and -independent pathways [42]. *IL24* functions as a potent anticancer cytokine through several mechanisms of tumor suppression. It can induce selective apoptosis and toxic autophagy in a broad spectrum of cancer cells without harming normal cells. *IL24* inhibits tumor growth by inducing endoplasmic reticulum (ER) stress and reactive oxygen species (ROS) production, blocking angiogenesis, sensitizing tumors to chemotherapy, and inhibiting the activities of proto-oncogenes like Src within tumor cells. Additionally, *IL24* stimulates secondary cytokines, which evoke anti-tumor immune responses [43,44]. In this study, we found that goose plasma and serum could increase the expression of *IL24* and regulate the JAK-STAT signaling pathway.

Metabolomic analysis revealed that many metabolites were significantly more abundant in goose plasma and serum. These metabolites may work together to inhibit the proliferation and migration of cancer cells. Oleoyl-L-carnitine was more abundant only in goose serum, whereas cucurbitacin D was more abundant in both goose serum and goose plasma. Oleoyl-L-Carnitine functions primarily as an acylcarnitine involved in the transport of long-chain fatty acids into mitochondria for energy production. As a conjugate of oleic acid and L-carnitine, it enables the fatty acid to cross the mitochondrial membrane, where it undergoes β-oxidation to generate ATP. Oleoyl-L-Carnitine plays a critical role in fatty acid metabolism and mitochondrial energy generation, as well as potentially modulating other transport and signaling pathways in cells [45]. L-carnitine shows promise as an anticancer agent by targeting the unique metabolic vulnerabilities of cancer cells, which include increased reliance on fatty acid oxidation (FAO) and a need for biosynthetic intermediates. Research indicates that L-carnitine can inhibit cancer cell growth, induce apoptosis, and modulate cellular bioenergetics, potentially through mechanisms involving histone acetylation and the regulation of mitophagy. This suggests it could be a valuable nutritional factor in managing cancer, particularly colorectal cancer (CRC) [46,47]. Some studies have found oleoyl-L-carnitine to be one of the crucial metabolites in pancreatic cancer patients [48]; however, its accumulation, particularly driven by obesity, has been implicated in promoting metastatic transformation in breast cancer models, suggesting it may influence cancer progression by altering lipid metabolism pathways [49]. Carnitine can impact tumor cell energy metabolism and reduce inflammation, potentially affecting cancer cell growth. Supplementation studies show that carnitine reduces cancer-related fatigue and may improve chemotherapy tolerance, though effects vary by cancer type. Carnitine transporters potentially serve as therapeutic targets or drug delivery routes in some cancers, enhancing the efficacy of chemotherapeutics [50,51].

Cucurbitacin D functions primarily as an anticancer and immunomodulatory compound. It exerts several cellular effects, particularly in cancer cells and immune cells. Cucurbitacin D inhibits cancer cell growth by arresting the cell cycle, often at the G1/S phase, through downregulation of cell cycle proteins such as cyclin D1, CDK4, and phosphorylated Rb protein. It induces apoptosis in cancer cells via activation of caspase-3 and cleavage of PARP, involving pathways such as JNK, STAT3, Akt, and NF-κB inhibition. Cucurbitacin D suppresses oncogenic signaling pathways, including PI3K/Akt, JAK/STAT, ErbB, and NF-κB pathways, reducing the expression of proliferative and metastatic proteins such as c-MYC and MMP9. It also upregulates tumor suppressor microRNAs and proteins such as p21, p27, and p53, contributing to cell cycle arrest and apoptosis. Its anticancer effect has been demonstrated in various cancer types, including cervical, breast, colorectal, ovarian, and hepatocellular carcinomas [52,53]. Cucurbitacin D functions as a potent inhibitor of multiple cancer-related signaling pathways, induces cell cycle arrest and apoptosis in cancer cells, and activates inflammatory responses in immune cells, making it a promising compound for cancer therapy and immunomodulation [54,55]. In this study, we found that levels of cucurbitacin D were significantly higher in goose serum and plasma than in chicken plasma and serum, and we speculated that cucurbitacin D may be a crucial metabolite responsible for the anticancer effects of goose blood.

Notably, the transcriptomic and metabolomic analyses in this study were constructed with distinct experimental purposes and comparison frameworks. The transcriptome focused on transcriptional alterations of SW1990 pancreatic cancer cells induced by goose serum/plasma, while metabolomics aimed to characterize differential endogenous metabolites between goose and chicken serum/plasma. Due to the inconsistent sample comparison systems, conventional pairwise correlation and joint pathway enrichment analysis were not applicable. Instead, this study adopted a logical two-step strategy: screening goose-specific candidate bioactive metabolites via metabolomics and exploring the underlying anticancer signaling mechanism via transcriptomics.

## 5. Conclusions

Goose blood holds natural medicinal value in China, and its promising anticancer potential has become a research hotspot amid advancing medical studies. Modern research has identified active components in goose blood, such as immunoglobulins, enzymes, and polysaccharides, that may fight cancer through multiple mechanisms: activating the immune system to enhance immune cell function against tumor cells, inducing tumor cell apoptosis, and inhibiting tumor angiogenesis. Goose serum and plasma share most of their components. Studies show that both can inhibit the proliferation and migration of SW1990 cells in a dose- and time-dependent manner, but serum induces both early and late apoptosis of SW1990 cells, while plasma induces only late apoptosis. Goose serum also inhibits human gastric cancer AGS cells and reduces cancer cell viability and migration in a dose- and time-dependent manner. RNA-seq analysis revealed key pathways involved: both plasma and serum upregulated genes such as RASSF6 in Hippo signaling and IL24 in JAK-STAT signaling and downregulated others such as IL17RE in IL-17 signaling and G6PD in cancer metabolism, which mediate their anticancer effects. Metabolomic analysis identified higher levels of certain metabolites in goose plasma and serum, including cucurbitacin D, which was present in both and is a known anticancer compound that may be key to goose blood’s anticancer effect, and serum-specific oleoyl-L-carnitine, which is linked to fatty acid metabolism and has a potential impact on cancer. These metabolites may synergistically inhibit cancer cell proliferation and migration.

## Data Availability

Data are available from the corresponding author upon reasonable request.

## Supplementary Materials

The following supporting information can be downloaded at https://www.mdpi.com/article/10.3390/biology15100788/s1.
