# Supplementary figures and images for: Transcriptomic and Metabolomic Profiling Reveals the Antiproliferative Mechanism of Goose Serum and Plasma in SW1990 Cells
Source: Biology (Basel). 2026 May 15;15(10):788. doi: 10.3390/biology15100788 (PMC13203157; doi:10.3390/biology15100788)

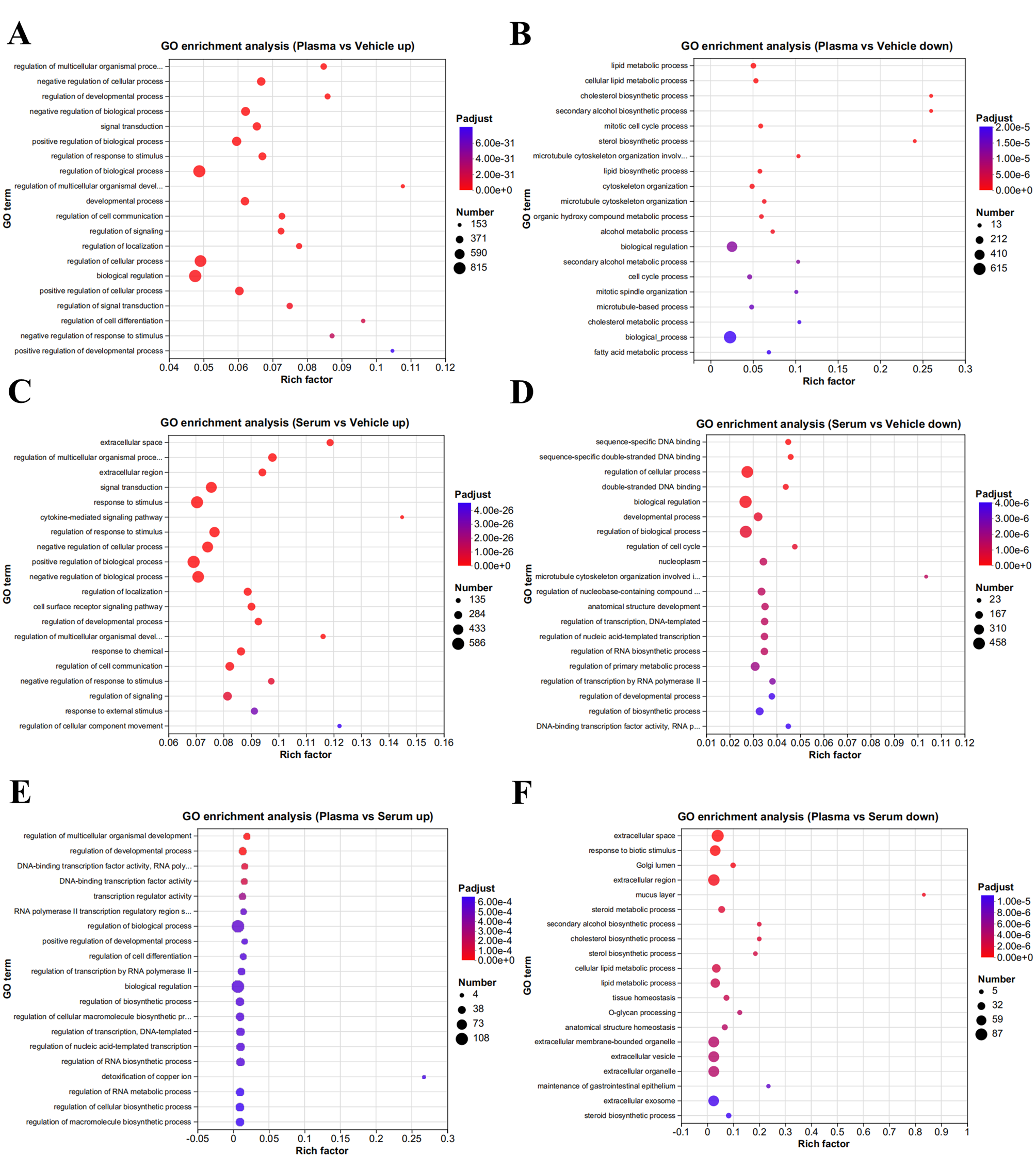

Supplement: Supplementary file 1 [file biology-15-00788-s001.zip › biology-4276550-supplementary/Figure S1.tif]
